# Supplementary material for: The effectiveness of care bundles for reducing caesarean section safely: A systematic review and meta-analysis
Source: PLoS One. 2025 Jun 13;20(6):e0326158. doi: 10.1371/journal.pone.0326158 (PMC12165343; doi:10.1371/journal.pone.0326158)
Supplement: S2 Table — (DOCX) [file pone.0326158.s002.docx]

**Supplementary File 2: Search Strategy and Results**

**CINAHL Plus (EBSCO): Searched 15-June-2024**

| **#Search** | **Search Terms** | **Records** |
| --- | --- | --- |
| S12 | ((TI bundl* OR ‘consensus bundle’ OR ‘safety bundle’ OR ‘care protocol’ OR ‘care algorithm’ OR ‘care protocols’ OR ‘care algorithms’ OR ‘clinical protocol’ OR ‘clinical algorithm’ OR ‘clinical protocols’ OR ‘clinical algorithms’ OR AB bundl* OR ‘consensus bundle’ OR ‘safety bundle’ OR ‘care protocol’ OR ‘care algorithm’ OR ‘care protocols’ OR ‘care algorithms’ OR ‘clinical protocol’ OR ‘clinical algorithm’ OR ‘clinical protocols’ OR ‘clinical algorithms’) AND (S7 OR S8 OR S9)) AND (S5 AND S6 AND S10): **Publication Date: 01-01-2000 to 12-06-2024** | **100** |
| S11 | ((TI bundl* OR ‘consensus bundle’ OR ‘safety bundle’ OR ‘care protocol’ OR ‘care algorithm’ OR ‘care protocols’ OR ‘care algorithms’ OR ‘clinical protocol’ OR ‘clinical algorithm’ OR ‘clinical protocols’ OR ‘clinical algorithms’ OR AB bundl* OR ‘consensus bundle’ OR ‘safety bundle’ OR ‘care protocol’ OR ‘care algorithm’ OR ‘care protocols’ OR ‘care algorithms’ OR ‘clinical protocol’ OR ‘clinical algorithm’ OR ‘clinical protocols’ OR ‘clinical algorithms’) AND (S7 OR S8 OR S9)) AND (S5 AND S6 AND S10) | 104 |
| S10 | (TI bundl* OR ‘consensus bundle’ OR ‘safety bundle’ OR ‘care protocol’ OR ‘care algorithm’ OR ‘care protocols’ OR ‘care algorithms’ OR ‘clinical protocol’ OR ‘clinical algorithm’ OR ‘clinical protocols’ OR ‘clinical algorithms’ OR AB bundl* OR ‘consensus bundle’ OR ‘safety bundle’ OR ‘care protocol’ OR ‘care algorithm’ OR ‘care protocols’ OR ‘care algorithms’ OR ‘clinical protocol’ OR ‘clinical algorithm’ OR ‘clinical protocols’ OR ‘clinical algorithms’) AND (S7 OR S8 OR S9) | 30682 |
| S9 | TI (bundl* OR ‘consensus bundle’ OR ‘safety bundle’ OR ‘care protocol’ OR ‘care algorithm’ OR ‘care protocols’ OR ‘care algorithms’ OR ‘clinical protocol’ OR ‘clinical algorithm’ OR ‘clinical protocols’ OR ‘clinical algorithms’) OR AB (bundl* OR ‘consensus bundle’ OR ‘safety bundle’ OR ‘care protocol’ OR ‘care algorithm’ OR ‘care protocols’ OR ‘care algorithms’ OR ‘clinical protocol’ OR ‘clinical algorithm’ OR ‘clinical protocols’ OR ‘clinical algorithms’) | 30682 |
| S8 | MH 'Clinical Protocol' | 0 |
| S7 | MH 'Care Bundle' | 0 |
| S6 | TI (cesarean OR caesarean OR caesarian OR cesarian OR ‘vaginal birth after caesarean’ OR VBAC OR ‘next birth after caesarean’ OR ‘trial of labour’ OR ‘trial of labor’ OR TOLAC) OR AB (cesarean OR caesarean OR caesarian OR cesarian OR ‘vaginal birth after caesarean’ OR VBAC OR ‘next birth after caesarean’ OR ‘trial of labour’ OR ‘trial of labor’ OR TOLAC) | 28,920 |
| S5 | (MH pregnan* OR birth OR intrapart* OR labour OR labor OR antenatal OR prenatal OR antepartum OR ante-natal OR ante-partum OR pre-natal) AND (S1 OR S2 OR S3 OR S4) | 240,089 |
| S4 | MH (pregnan* OR birth OR intrapart* OR labour OR labor OR antenatal OR prenatal OR antepartum OR ante-natal OR ante-partum OR pre-natal) | 205,052 |
| S3 | MH Childbirth | 11,422 |
| S2 | MH Obstetrics | 6,375 |
| S1 | MH Pregnancy | 231,924 |

AB: Abstract; MH: Major Subject Heading; TI: Title

**MEDLINE (OVID): Searched 15-June-2024**

| **#Search** | **Terms** | **Results** |
| --- | --- | --- |
| 1 | Pregnancy.sh. | 1014519 |
| 2 | Obstetrics.sh. | 25086 |
| 3 | Childbirth.sh. | 0 |
| 4 | (pregnan* or birth or intrapart* or labour or labor or antenatal or prenatal or antepartum or ante-natal or ante-partum or pre-natal).af. | 1507815 |
| 5 | 1 or 2 or 3 or 4 | 1517324 |
| 6 | (cesarean or caesarean or caesarian or cesarian or 'vaginal birth after caesarean' or VBAC or 'next birth after caesarean' or 'trial of labour' or 'trial of labor' or TOLAC).ab,ti. | 75879 |
| 7 | (bundl* or 'consensus bundle' or 'safety bundle' or 'care protocol' or 'care algorithm' or 'care protocols' or 'care algorithms' or 'clinical protocol' or 'clinical algorithm' or 'clinical protocols' or 'clinical algorithms').ab,ti. | 90124 |
| 8 | 5 and 6 and 7 (limited to 2000-2024) | **137** |

af: all fields; sh: Medical Subject Heading; ab.ti: Abstract.Title

**CENTRAL: Searched 15-June-2024**

| **#Search** | **Terms** (All text search) | **Records** |
| --- | --- | --- |
| #1 | pregnan* OR birth OR intrapart* OR labour OR labor OR antenatal OR prenatal OR antepartum OR ante-natal OR ante-partum OR pre-natal | 120987 |
| #2 | cesarean OR caesarean OR caesarian OR cesarian OR ‘vaginal birth after caesarean’ OR VBAC OR ‘next birth after caesarean’ OR ‘trial of labour’ OR ‘trial of labor’ OR TOLAC | 29073 |
| #3 | bundl* OR ‘consensus bundle’ OR ‘safety bundle’ OR ‘care protocol’ OR ‘care algorithm’ OR ‘care protocols’ OR ‘care algorithms’ OR ‘clinical protocol’ OR ‘clinical algorithm’ OR ‘clinical protocols’ OR ‘clinical algorithms’ | 163935 |
| #4 | #1 AND #2 AND #3 | 3081 |
| #5 | Limit to trials and year 01-Jan-2000 to 15-Jun-2024 | **1840** |

**Embase: Searched 15-June-2024**

| **#Search** | **Terms** | **Records** |
| --- | --- | --- |
| #9 | #8 AND 2000-2024:py | **5427** |
| #8 | #3 AND #4 AND #7 | 5845 |
| #7 | #5 OR #6 | 1033292 |
| #6 | bundl* OR (consensus AND bundle) OR (safety AND bundle) OR (care AND protocol) OR (care AND algorithm) OR (care AND protocols) OR (care AND algorithms) OR (clinical AND protocol) OR (clinical AND algorithm) OR (clinical AND protocols) OR (clinical AND algorithms):ab.ti | 1033292 |
| #5 | care AND bundle:de OR (clinical AND protocol:de) | 153437 |
| #4 | cesarean OR caesarean OR caesarian OR cesarian OR (vaginal AND birth AND after AND caesarean) OR vbac OR (next AND birth AND after AND caesarean) OR (trial AND of AND labour) OR (trial AND of AND labor) OR tolac:ab,ti | 175047 |
| #3 | #1 OR #2 | 1905355 |
| #2 | 'pregnancy'/mj OR 'obstetrics'/mj OR 'childbirth'/mj | 229841 |
| #1 | pregnan* OR 'birth'/exp OR birth OR intrapart* OR 'labour'/exp OR labour OR 'labor'/exp OR labor OR antenatal OR 'prenatal'/exp OR prenatal OR antepartum OR 'ante natal' OR 'ante partum' OR 'pre-natal' | 1890863 |

ab.ti: Abstract.Title; de: Indexed term; exp: explode using Emtree terms

Top of Form
