# Supplementary material for: The effectiveness of care bundles for reducing caesarean section safely: A systematic review and meta-analysis
Source: PLoS One. 2025 Jun 13;20(6):e0326158. doi: 10.1371/journal.pone.0326158 (PMC12165343; doi:10.1371/journal.pone.0326158)
Supplement: S3 Table — (DOCX) [file pone.0326158.s003.docx]

**Supplementary File 3: PRESS Checklist complete**

**Review Title:** The effectiveness of care bundles for reducing caesarean section safely: A systematic review and meta-analysis.

**Name of PRESS Reviewer:** Dr D O’Malley

**Role:** Midwife, academic and researcher

**Date of PRESS review:** 30^th^ April 2024

**PICOS:**

P - Women who are pregnant or in labour

I - Care bundles (as per IHI definition) developed for the safe reduction of caesarean section.

C - No care bundle/standard care

O - Maternal and neonatal outcomes

S - Randomised and non-randomised studies

**Checklist items**

| **Category** | **Item** | **Y/N/NA** | **Comment (if relevant)** |
| --- | --- | --- | --- |
| Translation of the research question | Does the search strategy match the research question/PICO? | Y | Care bundles that incorporate a preconceptual element will not be captured |
|  | Are the search concepts clear? | Y |  |
|  | Are there too many or too few PICO elements included? | N |  |
|  | Are the search concepts too narrow or too broad? | N |  |
|  | Does the search retrieve too many or too few records? (Please show number of hits per line) | N | P – 1,451,594  C – 81,287  I – 193,289  Combined – 39 (CINAHL) |
|  | Are unconventional or complex strategies explained? | NA |  |
| Boolean and proximity operators | Are Boolean or proximity operators used correctly? | Y |  |
|  | Is the use of nesting with brackets appropriate and effective for the search? | Y |  |
|  | If NOT is used, is this likely to result in any unintended exclusions? | NA |  |
|  | Could precision be improved by using proximity operators (e.g., adjacent, near, within) or phrase-searching instead of AND? | N |  |
|  | Is the width of proximity operators suitable (e.g., might adj5 pick up more variants than adj2)? | NA |  |
| Subject headings | Are the subject headings relevant? | Y |  |
|  | Are any relevant subject headings missing, e.g., previous index terms? | N |  |
|  | Are any subject headings too broad or too narrow? | N |  |
|  | Are subject headings exploded where necessary and vice versa? | Y |  |
|  | Are major headings (“starring” or restrict to focus) used? If so, is there adequate justification? | Y |  |
|  | Are subheadings missing? | N |  |
|  | Are subheadings attached to subject headings? (Floating subheadings may be preferred) | N/A |  |
|  | Are floating subheadings relevant and used appropriately? | NA |  |
| Text word searching | Does the search include all spelling variants in free text (e.g., UK versus US spelling)? | Y |  |
|  | Does the search include all synonyms or antonyms (e.g., opposites)? | NA |  |
|  | Does the search capture relevant truncation (i.e., is truncation at the correct place)? | Y |  |
