# Supplementary material for: The effectiveness of care bundles for reducing caesarean section safely: A systematic review and meta-analysis
Source: PLoS One. 2025 Jun 13;20(6):e0326158. doi: 10.1371/journal.pone.0326158 (PMC12165343; doi:10.1371/journal.pone.0326158)
Supplement: S4 Table — (DOCX) [file pone.0326158.s004.docx]

**Supplementary File 4: Details of the care bundle interventions**

**Bell 2017; Callaghan-Koru 2021a; 2021b**

The Council on Patient Safety in Women’s Health Care Safe Reduction of Primary Cesarean Births <https://saferbirth.org/wp-content/uploads/R2_AIM_SRPCB_PSB-3.pdf>

*Readiness — Every Unit*

- Develop provider, patient community and unit culture that values, promotes, and supports spontaneous onset and progress of labour and vaginal birth and understands the risks for current and future pregnancies of caesarean birth without medical indication.
- Provide education to pregnant people and families related to their options for labour and birth throughout the perinatal care cycle, with an emphasis on informed consent, and shared decision-making.
- Facilitate multidisciplinary education to healthcare team members on approaches which maximize the likelihood of vaginal birth, including assessment of labour, methods to promote labour progress, labour support, coping mechanisms, and pain management (both pharmacologic and non-pharmacologic), and shared decision-making to all providers and staff that provide care to pregnant and postpartum people.
- Training on trauma-informed care and health care team member biases to enhance high-quality, equitable outcomes.

*Recognition & Prevention — Every Patient*

- Implement standardized admission criteria, triage management, education, and support for people presenting in spontaneous labour.
- Ensure availability and offer a range of standard techniques of pain management and comfort measures that promote labour progress and prevent dysfunctional labour.
- Utilize standardized methods in the assessment of the fetal heart rate status, including interpretation and documentation and encourage evidence-based positioning and patient movement in labour.
- Implement protocols for timely identification of specific conditions, such as active herpes and breech presentation, for patients who can benefit from proactive intervention before labour to reduce the risk for caesarean birth.
- Implement standardized approaches to promote evidence-based interventions for conditions such as macrosomia, low-lying placenta, and oligohydramnios.

*Response — Every Event*

- Ensure availability of clinicians, staff, and resources to maintain appropriate ongoing labour assessment and support and respond to labour process disruptions and emergencies.
- Uphold comprehensive standardized induction scheduling with shared decision-making, planning, and preparation of patients undergoing induction.
- Utilize standardized evidence-based labour algorithms, policies, and techniques, which allow for prompt recognition and treatment of dystocia and are consistent with the diagnosis of labour dystocia criteria.
- Adopt policies that outline standard responses to abnormal fetal heart rate patterns and uterine activity to avoid unnecessary intervention and maintain high-quality neonatal outcomes.
- Provide via clinician training, skill development, or referral expertise and techniques to lessen the need for abdominal delivery, such as breech version, instrumented delivery, and twin delivery protocols.

*Reporting and Systems Learning — Every Unit*

- Perform regular multidisciplinary reviews of indications for caesarean births to determine alignment with established standards to identify systems issues and variations in provider performance.
- Monitor appropriate metrics and balancing measures, including maternal and newborn outcomes resulting from changes in labour management strategies, with disaggregation by race and ethnicity due to known disparities in rates of caesarean birth.
- Establish a culture of multidisciplinary planning, huddles, and post-event debriefs for unplanned caesarean births, which identify success, opportunities for improvement, and action planning for future events.

**Fan 2021**

1. Confidential audit-and-feedback of obstetrician rates every 3 months
2. Provider-centred educational rounds
3. Physician-patient decision aid
4. Patient-centred resources.

**Garpiel 2018**

Selected practices were structured in a bundle framework called 5-Ps of Safe Second-Stage Labor Care.

1. Patience: Delay pushing up to 2hrs after full dilatation or until urge to push, or fetal head at the introitus with epidural; support spontaneous pushing efforts; lengthen pushing duration of progress and fetal tolerance is evident
2. Positioning: Promote upright, lateral, and non-supine maternal positions to facilitate maternal comfort and fetal progress to birth, support and assist labouring woman to change positions every 15 to 30 minutes, use labour bed features and position aids, for example, a peanut ball, and avoid routine lithotomy positioning during pushing, unless maternal or fetal conditions warrant an expeditious birth
3. Physiologic resuscitation: manage uterine tachysystole and promote fetal recovery during stressful contractions; decrease or discontinue exogenous oxytocin at second-stage onset to modify physiologic labour contraction patterns and prevent tachysystole, if tachysystole occurs, decrease oxytocin rate by half if FHR pattern is Category I or discontinue the oxytocin if FHR pattern is Category II or III, and avoid increasing oxytocin to facilitate pushing to expedite birth
4. Progress: Evaluate and communicate at regular time intervals birth progress and maternal–fetal tolerance to enable team members to develop a collaborative plan for a safe birth
5. Preventing urinary harm: avoid indwelling urinary catheters during labour, assess bladder status and encouraging women to void before placing an epidural, offer a bedpan or use intermittent catheterization to empty her bladder if determined to be over distended, assess bladder status throughout labour at least every 2 to 4 hours, and consider performing an intermittent catheterization immediately prior to onset of maternal pushing

**Miazga 202o; 2021a; 2021b**

1. Educational rounds for health care providers
2. A TOLAC physician-patient decision aid
3. Patient educational tools (educational handouts, videos, and posters)

**Ryan 2012**

Project Primip:

1. Supportive, one-to-one midwifery care
2. Defined and documented diagnosis of labour
3. Documented management plan at 2-hourly intervals
4. Extended Birth Suite coverage by Consultant Obstetricians.

This bundle of care is supported by: (i) strict adherence to the ‘Care in Labour and Birth’ guideline; (ii) revision of midwifery resource allocation; and (iii) education and communication.

**Telfar 2021**

1. A shared-decision labour triage guide - evidence-based graphic algorithm poster displayed in each triage/labour room and utilized by the staff and patient for education, individualization of care, and choosing a plan of care
2. An early labour walking path - a mapped out walk around the hospital encouraging movement with specific stations to visit which included exercises and links to music and guided meditations
3. A labour partograph
4. A pre-caesarean checklist. A labour partogram and pre-caesarean checklist were provided to guide and standardize evidence-based practice and guide conversations and decision-making with patients. The tool was available for use by staff and simultaneously the QI initiative leader used the tool to conduct chart reviews as a mechanism of audit and feedback for the team

**Tolcher 2016**

1. Dedicated obstetric attending presence on the unit 24 hours a day
2. Communication training for all providers including physicians, certified nurse midwives, and nurses
3. Mandatory use of a labor partogram
4. Mandatory fetal heart rate tracing interpretation training (K2 Medical Systems)
5. Regular feedback on individual provider Adverse Obstetric Index
