# Supplementary material for: The effectiveness of care bundles for reducing caesarean section safely: A systematic review and meta-analysis
Source: PLoS One. 2025 Jun 13;20(6):e0326158. doi: 10.1371/journal.pone.0326158 (PMC12165343; doi:10.1371/journal.pone.0326158)
Supplement: S6 Fig — (DOCX) [file pone.0326158.s006.docx]

**Supplementary File 6: Additional Forest Plots**

**Figure S1.1: Subgroup analysis Nulliparous versus Multiparous**

**
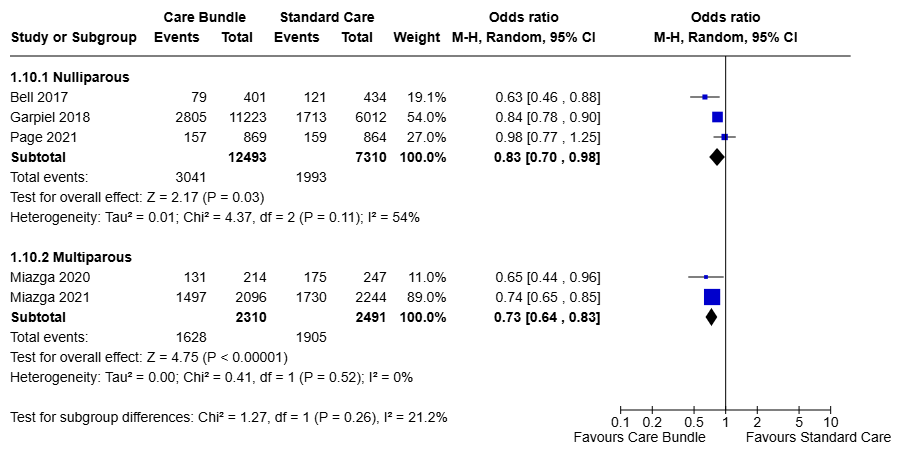
**

**Figure S1.2: Subgroup analysis duration up to 6 months versus > 6 months**


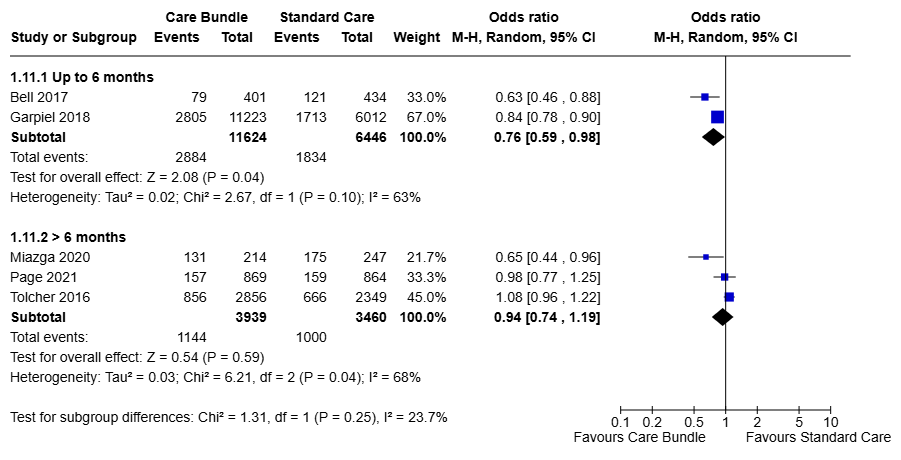


**Figure S2: Emergency CS**

**
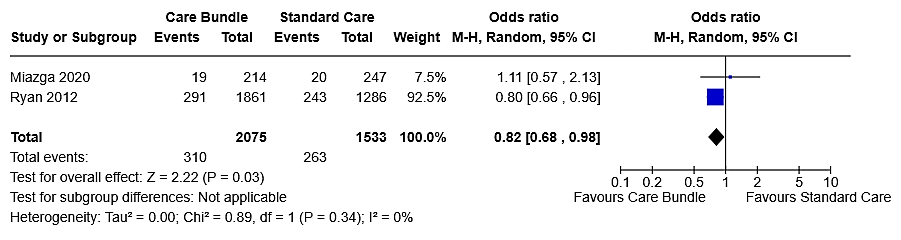
**
